# Commentary: Physical time within human time

**DOI:** 10.3389/fpsyg.2023.1094736

**Published:** 2023-06-05

**Authors:** Elisa Paganini

**Affiliations:** Dipartimento di Filosofia “Piero Martinetti”, Università degli Studi di Milano, Milano, Italy

**Keywords:** time experience, illusion, hallucination, block-universe, local past/present/future

## 1. Introduction

The aim of this contribution is to compare the interesting and challenging theoretical proposals of Gruber et al. (2022) on the one side, and of Buonomano and Rovelli (2021) on the other. A problem they both address is whether our experience of time (characterized by the recognition of the flow of time and the impermanence of the present) is a veridical or illusory representation of the physical reality of the Universe. My claim is that the two theoretic proposals are adopting different notions of illusions and that a better understanding of each of the two notions would be useful to further develop the proposals.

The theoretical positions proposed in the two essays are divergent: Gruber et al. (2022) claim that physical theories stipulate a universe without past, present and future (i.e., a Block Universe), and that our temporal experience (which instead discriminates between past, present and future) is illusory; Buonomano and Rovelli (2021) argue for a more complex and multilayered view of time: on the one side they acknowledge that past, present and future do not feature fundamental physics, not being “universal,” but they are open to their being “locally” instantiated and our perception not necessarily being illusory about local reality. I am not going to compare the reasons in support of the two cosmological theories; I am adopting a philosophical perspective instead, my aim is to discuss the two philosophical notions of illusion that underlie the two different theoretical approaches, with a view to showing that each notion of illusion proposes interesting and pressing challenges to the respective theoretical proposal.

## 2. Illusion as cognitive or perceptual add-on

Our commonsense notion of illusion is connected to cases in which our perceptions are deceived by certain aspects of reality. For instance, when viewing a *tromp l'oeil*, we perceive a three-dimensional reality, even though the image is actually two-dimensional, or, in the case of the Müller-Lyer illusion, we see lines of different lengths, while we are confronted with lines of the same length. In such cases, features of what is perceived (as the perspective in the *tromp l'oeil* or the bracketing lines in the Müller-Lyer illusion) cause our perception to be deluded. It is important to realize that this is not the notion of illusion Gruber et al. (2022) have in mind: they claim that “the term illusion refers to a perception which has no basis in reality” and they refer to illusion as a “perceptual or cognitive add-on” (Gruber et al., 2022, p. 3). If I understand them correctly, the idea is that the mind adds to what is perceived, allowing for the experience of the flow of time, which does not have any support in external reality. The experience of time—as presented by Gruber et al. (2022)—seems more like a hallucination or a mental projection (produced by certain characteristics of the subject independently of the surrounding reality) than an illusion.

This approach is in line with recent B-theorist philosophers' attacks on the A-theorist philosophers' claim that our perception of time reflects or is determined by an objective temporal flux. B-theorists argue that our experience of time is unreliable: for example, it is contended that perception mistakes fast discontinuous change for continuous change (Paul, 2010); moreover, it is claimed that temporal experience cannot represent an objective flow of time considered as a condition of any perception (Prosser, 2013); and, it is maintained that the feeling of the passage of time “is not a worldly representational feature of our experience” (Torrengo, 2017, p. 185). To these observations, Gruber et al. (2022) add that the IGUS experiment shows that “the actual “moving present” is a dynamistic illusory experience that is more related if not identical to the experience of “moving” which is itself illusory (Gruber et al., 2022, p. 4).

When confronted with all these arguments in support of our unreliable perceptions, the first question which arises is: is there any role for hallucinatory time experience in a Block universe? Is our hallucinatory experience useless? Why do we have these deceptive experiences? Gruber et al. (2022) writes that the illusory system allows humans to be “more functional” and to develop “adaptive behavior” (Gruber et al., 2022, p. 1 and 11). This is an interesting observation which differentiates Gruber et al.'s proposal from others in the literature. In my opinion, this interesting claim requires some explanation. First, it is not clear what the words “functional” and “adaptive” mean in a Block universe. In a changing perspective, a system is *functional* when it reacts adequately to certain stimuli and the reaction happens in a changing time; but what is a functional system in a Block universe? i.e., what is a Block-functional system? Moreover, in a changing perspective, species *adapt* to a certain environment when they develop features allowing for survival, where the notions of “developing features” and “survival” are interpreted in a changing time; but what is adaptation in a Block universe? i.e., what is Block-adaptation? Once these notions are explained, it would be useful to clarify why the illusory experiences—and not the veridical ones—allow humans to be Block-functional and Block-adaptive.

## 3. Illusion as misperception

If it is admitted—as Buonomano and Rovelli (2021) interestingly suggest—that past, present and future are “locally”—but not “universally”—instantiated, it is worth considering whether our temporal experience simply represents the local reality as it is. Buonomano and Rovelli's proposal does not require that the temporal experience be simply representational. They maintain that certain objective characteristics of the universe (as entropy gradients, traces of the past and the macroscopic under-determinacy of the future) “underlie the brain's ability to produce a subjectively vivid and richly structured “flow of time” (Buonomano and Rovelli, 2021). The idea is therefore that there are intrinsic “local” characteristics of the universe which are somehow interrelated to subjective experiences.

The proposal is reminiscent of Locke's secondary qualities (Locke, 1690). Locke distinguishes between primary and secondary qualities: primary qualities are properties of the external reality represented as they are in themselves, secondary qualities are properties of the external reality which have the “power to produce various sensations in us” (Locke, 1690, II, viii, 10). Locke's distinction is not between an external and an internal standpoint on the self^1^, it is instead between two different ways in which objective mind-independent properties may affect humans' sense organs. Locke's idea is that secondary properties are not represented as they are, they interact with our perceptual organs to produce certain subjective experiences in us. The paradigms of secondary qualities presented by Locke are color, smell, taste, and sound. For example, the physical properties which produce the experience of taste in us do not have these experienced qualities in themselves, the experience of taste is the way we apprehend physical properties. Under this interpretation, the ability to experience taste, even if not simply representational, allows us to keep track of certain characteristics of the external world (i.e., the experience of taste allows us to keep track of certain physical characteristics of food).

Along Locke's ideas, I interpret Buonomano and Rovelli's original proposal of the subjective experience of the passage of time allowing us to keep track of certain external characteristics of the “local” reality, without necessarily representing them as they are. As Buonomano and Rovelli acknowledge, their hypothesis gives rise to many questions for investigation by physics and neuroscience; and—I add—also philosophy is useful in this respect. In my opinion, it is within this approach that A-theorist philosophers may defend their tenet against the B-theorist who considers the experience of the passage of time as an illusion or a hallucination, allowing instead our experience of the flow of time to be, even though not simply representational, able to monitor properties independent of the subjects themselves.

To further develop the research, I believe it useful to individuate the phenomenal characteristics which interact with the objective local flow of time, thus making it possible to further analyze any case of misperception. Considering again the case of taste, we may misperceive the taste of a particular ingredient when it is covered by the taste of another (therefore our perception of the covered ingredient may be illusory because of external factors), but it may also happen that the subject is unable to perceive an objective taste because of illness (in such a case an internal factor may create a hallucination or mental projection). In the case of time, once the physical data of the objectively local temporal passage and the phenomenal corresponding experience have been individuated, it will be possible to establish the causes of misperception in factors either external or internal to the subject. Far from constituting a reason for being skeptical about the passage of time, temporal experiences may keep track of external phenomena and, when they do not, they constitute experimental data for distinguishing between cases in which the illusion depends on external factors from cases in which it depends on internal factors.

## Author contributions

The author confirms being the sole contributor of this work and has approved it for publication.

## Footnotes

^1^A distinction which may be found in Fechner (1851), II, 362 translated in (Heidelberger, 2004, p. 77–78) between an internal perspective on the self [“a standpoint of inner self-phenomena”] and an external perspective on it [“an external standpoint”].
